# CO_2_ Laser and Topical Fluoride Therapy in the Control of Caries Lesions on Demineralized Primary Enamel

**DOI:** 10.1155/2015/547569

**Published:** 2015-03-22

**Authors:** R. A. Valério, C. T. Rocha, R. Galo, M. C. Borsatto, M. C. P. Saraiva, S. A. M. Corona

**Affiliations:** ^1^Clinical Pediatric Dentistry Department, Ribeirão Preto School of Dentistry, São Paulo University, Café Avenue, Monte Alegre, 14040-904 Ribeirão Preto, SP, Brazil; ^2^Restorative Dentistry Department, Ribeirão Preto School of Dentistry, São Paulo University, Café Avenue, Monte Alegre, 14040-904 Ribeirão Preto, SP, Brazil

## Abstract

This study evaluated the effect of CO_2_ laser irradiation and topical fluoride therapy in the control of caries progression on primary teeth enamel. 30 fragments (3 × 3 × 2 mm) from primary canines were submitted to an initial cariogenic challenge that consisted of immersion on demineralizing solution for 3 hours and remineralizing solution for 21 hours for 5 days. Fragments were randomly assigned into three groups (*n* = 10): L: CO_2_ laser (*λ* = 10.6 *μ*m), APF: 1.23% acidulated phosphate fluoride, and C: no treatment (control). CO_2_ laser was applied with 0.5 W power and 0.44 J/cm^2^ energy density. Fluoride application was performed with 0.1 g for 1 minute. Cariogenic challenge was conducted for 5 days following protocol previously described. Subsurface Knoop microhardness was measured at 30 *μ*m from the edge. Obtained data were subjected to analysis the variance (ANOVA) and Duncan test with significance of 5%. It was found that the L group showed greater control of deciduous enamel demineralization and were similar to those of APF group, while being statistically different from C group (*P* ≤ 0.05) that showed the lowest microhardness values. It was concluded that CO_2_ laser can be an additional resource in caries control progression on primary teeth enamel.

## 1. Introduction

Application of fluoride compounds has been used to control dental caries in primary teeth under different forms [1, 2] and different concentrations [3]. The mechanism of fluoride interferes in the process of mineral loss, promoting inhibition of demineralization, and enhancement dental substrate remineralization [4]. The ability of acidulated phosphate fluoride (APF) to become the primary teeth and more acid-resistant when exposed to cariogenic challenge was evidenced by Castellan et al. 2007 [1]. However, for an effective fluoride action controlling demineralization, it must be constantly in the oral cavity [5].

Higher incidence of dental caries in primary teeth associated with rapid progression of these lesions due to lower mineral content [6] leads to early loss of these teeth [7], factors that encourage more studies to improve existing preventive treatments and to evaluate innovative techniques such as CO_2_ laser irradiation [8, 9].

CO_2_ laser irradiation is more appropriate to dental enamel because it produces radiation in the infrared region (9.3, 9.6, 10.3, and 10.6 *μ*m) that coincides closely with some of apatite absorption bands, mainly phosphate and carbonate group absorption [10]. Therefore, higher effectiveness in caries prevention could be achieved with lower occurrence of harmful effects to dental tissues [10]. Using this laser, energy is absorbed in few micrometers of the external enamel surface and converted into heat, causing loss of carbonate from mineral and fusion of hydroxyapatite crystals, reducing the interprismatic spaces [11]. Furthermore, it increases its acid resistance, decreasing the mineral reactivity and promoting caries-preventive effect [9].

The CO_2_ laser may control caries progression in permanent [12] and bovine enamel [13] when compared to fluoride compounds [14]. The efficacy of this laser in caries control on demineralized primary enamel was also previously evaluated by Tagliaferro et al. 2006 [8] and da Silva Tagliaferro et al. 2009 [9]. However, in these studies, laser was applied on sound enamel. There are no studies in the literature evaluating the effect of CO_2_ laser in previously demineralized primary enamel, simulating a patient with high cariogenic challenge and high caries risk.

As creation of an acid-resistant surface seems to be a promise in the control of caries lesions, the aim of this study was to evaluate* in vitro* the effect of CO_2_ laser irradiation and topical fluoride therapy in control of caries progression on enamel of primary teeth by subsurface microhardness analysis.

## 2. Material and Methods

### 2.1. Experimental Design

The factor under investigation was surface treatment at 3 levels: L: CO_2_ laser irradiation; APF: 1.23% acidulated phosphate fluoride; C: no treatment (control). The sample consisted of 30 fragments of human primary enamel distributed among three surface treatments (*n* = 10), according to a randomized and complete block design. The quantitative response variable was the subsurface Knoop microhardness (KHN) of the substrate subjected to the chemical demineralization* in vitro*.

### 2.2. Ethical Aspects

This research was approved by the Ethics in Research Committee of the School of Dentistry of Ribeirão Preto, University of São Paulo (Process number 2010.1.1373.58.9). Freshly extracted sound primary canines were obtained from Human Tooth Bank of the same institution.

### 2.3. Selection and Preparation of Samples

Primary teeth were hand scaled and cleaned with water/pumice slurry, in rotating bristle brushes at low speed (N270, Dabi Atlante, Ribeirão Preto, SP, Brazil) to remove calculus and surface-adhered debris and stored in 0.1% thymol solution. The absence of cracks, hypomineralization, and hypoplasia was confirmed under an ×20 magnifier (Leica S6 D Stereozoom, Mycrosystems Leica AG, Switzerland) and teeth with structural defects were discarded. Afterwards, the selected teeth were sectioned in the cement-enamel junction in precision cutter water-cooled (Isomet 1000, Buehler, Lake Bluff, IL, USA), to separate the root and coronal portions. The buccal surface of each tooth was sectioned to obtain a fragment of enamel measuring 3 × 3 × 2 mm.

The fragments were fixed in acrylic resin blocks using melted wax (Wax Sculpture Fixed Prosthodontics, Aspheric Chemical Industry Ltda., São Caetano do Sul, SP, Brazil) with the subsurfaces facing the external environment. The subsurfaces were then flattened with #1200-grift silicon carbide paper in a water-cooled polishing machine (Politriz, DP-9U2, Struers A/S, Copenhagen, Denmark) (Hermes Abrasives Ltd., VA, USA) and polished with 0.3 *μ*m alumina paste (Arotec S/A Ind. Com, SP, Brazil) by felt polisher (ATM, Altenkirchen, Germany) [15]. In order to obtain a sample of patterned fragments, three readings were performed on the side of the fragments (subsurface) 30 *μ*m from the edge and 100 *μ*m of each other through a microhardness tester HMV-2000 (Shimadzu Corporation, Kyoto, Japan) with a diamond indenter for Knoop hardness (KHN) under 25 g load for 5 seconds [11]. The three readings were averaged and used as the microhardness value of each fragment. Specimens with microhardness values 20% above or below the mean value of all fragments were discarded [16]. Thirty fragments of primary enamel were selected based on initials Knoop hardness values of its fragments lateral side.

### 2.4. Initial Cariogenic Challenge

For obtaining initial microscopic lesions of standardized white spot lesion, simulating patients with high caries activity, an artificial caries challenge was performed in all fragments. The specimens were repositioned with the buccal surface facing the external environment in resin blocks and fixed with wax. All surfaces except the buccal were covered with melted wax and stored individually in plastic containers. The initial cariogenic challenge was performed during 5 days according to the protocol proposed by Argenta et al. 2003 [17]. Artificial caries lesions were produced by immersion of the fragments in demineralizing solution (pH 4.6) for 3 hours and remineralizing solution (pH 7.0) for 21 hours at 37°C. After the artificial carious lesions formation, the specimens were kept in humidity for 2 days at 4°C.

### 2.5. Surface Treatment

According to a complete block design and randomized, the specimens were divided according to treatment in three groups (*n* = 10): L: CO_2_ laser, APF: 1.23% acidulated phosphate fluoride, and C: no treatment (control).

The CO_2_ laser with *λ* = 10.6 *μ*m (PC 015-D CO_2_ Laser System, Shanghai JueHua Laser Tech. Development Co., Ltd., Shanghai, China) was applied in ultrapulsed mode, 0.5 W average power, 0.44 J/cm^2^ energy density measured with Power Meter (FieldMax II-TOP, Coherent Inc., Santa Clara, USA), 100 *μ*s pulse duration, 0.001 sec interval between pulses, 0.4 mm beam diameter on the substrate surface, where the operator kept the laser tip perpendicularly to the substrate with distance tip/substrate of 4 mm [18] for 20 sec. Parameters used in the present study were able to produce only chemical and structural modification on primary enamel, without causing surface damage or tissue removal. After irradiation, the samples were kept in artificial saliva at 37°C for 24 hours. These were the components of artificial saliva, the reagent (213 mg of CaCl_2_·H_2_O, 738 mg of KH_2_PO_4_, 1.114 mg of KCl, 381 mg of NaCl, 12 g of Tris, 2.2 g of gastric mucin, and qsp 1 liter) weighed on an analytical balance (AB204-S/FACT, Mettler Toledo, Columbus, OH, USA) and subjected to agitation, adjusting the pH to 7.0.

0.1 g of 1.23% acidulated phosphate fluoride gel (DFL Industry, Rio de Janeiro, RJ, Brazil, pH 3.6) was weighed on analytical balance (AUW220D, SPLABOR, Presidente Prudente, SP, Brazil) and applied to the dry surface deciduous enamel using microbrush (KG Sorensen, Cotia, SP, Brazil). After 1 minute [19], the specimens were washed with deionized water for 10 seconds, dried with absorbent paper, and after stored in artificial saliva at 37°C for 24 hours.

The control group did not receive any treatment, being kept in artificial saliva at 37°C for 24 hours.

### 2.6. Cariogenic Challenge Postsuperficial Treatment

The samples were replaced in plastic containers and all surfaces, except for the treated surface, and were covered with melted wax. The same pH cycling that was applied before the laser or the fluoride treatment was repeated 5 times, at a rhythm of one per day, in order to simulate the conditions of cariogenic severe challenge.

### 2.7. Microhardness Test

After cariogenic challenge period, specimens were sectioned longitudinally and fixed with melted wax and their internal side (sectional) was left exposed and polished in a polishing machine (DP-9U2; Struers S/A, Copenhagen, Denmark). After polishing, specimens were observed under an optical microscope to verify the superficial smoothness and were subjected to ultrasonic cleaning (Dabi Atlante, Ribeirão Preto, SP, Brazil) for two minutes to remove the debris. Then, impressions were made in one of the hemisections, keeping the long axis of the diamond indenter parallel to the external surface of the enamel using a static load of 25 g for 5 sec [1]. Three measurements were performed at the center of the fragment, with 100 *μ*m in distance from one another, 30 *μ*m from the edge, totalizing 3 indentations per specimen. The readings were averaged and used as the microhardness value of each slab, using a microhardness tester HMV-2000 (Shimadzu Corporation, Kyoto, Japan).

**Figure 1:**
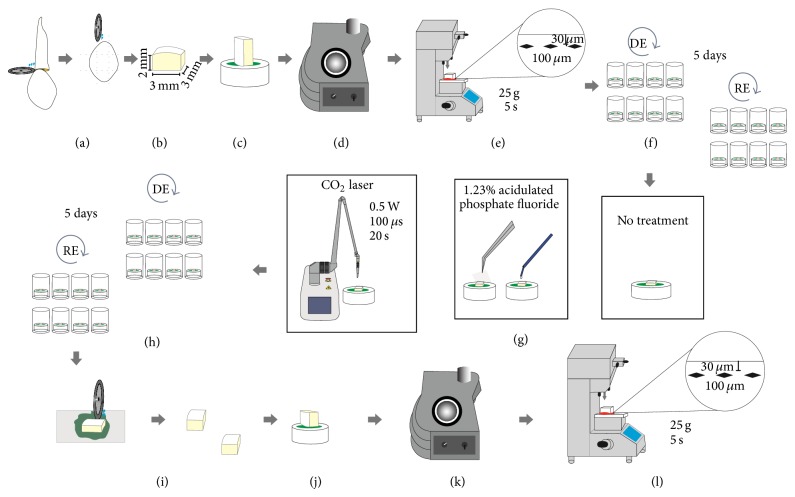
Schematic design of the methodology presented. (a) Section of the teeth. (b) Obtaining fragments. (c) Fixation of specimens in resin blocks. (d) Planning and polishing the enamel surface. (e) Selection of specimens. (f) Initial cariogenic challenge. (g) Surface treatments. (h) Cariogenic challenge after surface treatment. (i) Section of the fragments. (j) Fixing the fragments into blocks of acrylic resin. (k) Polishing the enamel surface. (l) Microhardness evaluation.

The protocol used in this study is shown in Figure 1.

### 2.8. Statistical Analysis

The mean values of microhardness of each specimen were analyzed and showed a normal distribution and homogeneity of variance. Thus, analysis of variance (ANOVA) was employed. The Duncan test was used to investigate differences between the mean of surface treatment factor using SPSS 12.0 for Windows (SPSS Inc., Chicago, IL, USA) with a significant level of 5%.

## 3. Results

The results showed that microhardness of subsurface treatments performed on primary teeth enamel was statistically different (*P* ≤ 0.05), as shown in Table 1.

Duncan test showed that surface treatment with CO_2_ laser showed the highest microhardness values (KNH) on primary teeth enamel, but it was not statistically different from 1.23% acidulated phosphate fluoride application. However, a statistically significant difference from the control group that presented the lowest microhardness values was found.

## 4. Discussion

Acidulated phosphate fluoride [1, 2] and CO_2_ laser radiation [8, 9] have been used to prevent caries in primary teeth in order to interfere the balance of deremineralization. The effects of laser irradiation on the tissue are closely related to wavelength, absorption of laser light by the irradiated tissue, laser power, emission mode, energy density, and frequency [20, 21].

CO_2_ laser is responsible for increasing acid resistance on irradiated enamel [1, 8, 9]. On the other hand, fluoride is able to incorporate on dental substrate, preventing the development of carious lesions, inhibiting enamel demineralization, and enhancing remineralization through minerals gain [3].

In this study, surface treatment with CO_2_ laser in primary enamel was statistically similar to 1.23% acidulated phosphate fluoride. The probable reason for the increased acid resistance of the primary enamel after CO_2_ laser treatment is a consequence of thermal effect [22, 23]. Heating of tooth surface results in structural and chemical alterations in the irradiated dental substrates with melting point of hydroxyapatite [24], regarding calcium [25, 26] and phosphorus loss [27], calcium and phosphorus concentration on the surfaces [28], and alterations in organic matrix [29].

Thermal variations produced by using the CO_2_ laser on enamel promote reduction of water and carbonate content [22] which is converted into phosphate followed by protein decomposition at temperatures of 100–650°C, thermal recrystallization (650 and 1.100°C), and destructive phenomena such as melting of hydroxyapatite (>1.100°C) [30]. CO_2_ laser may decrease dental permeability and hinder diffusion of acids, due to the surface sealing [31], reducing the demineralization of dental structure [10]. The enamel irradiated using high energy densities revealed nonhydroxyapatite phases, apparently similar to tri- and tetracalcium phosphates [32].

The thermal effects are responsible for changes in the irradiated tooth surfaces while they may differ from the temperature observed at pulp chamber, due to the support structures present around the teeth and the blood flow of the pulp tissue; this heat could be dissipated [33, 34]. The pulp temperature increase, related to the use of high power lasers, is based on the amount of energy applied and therefore, the exposure time is fundamental. High energy densities in short periods of time cause less pulp damage [35], since the thermal relaxation is inversely proportional to the square of the irradiated volume [33].

The low thermal conductivity of the enamel and the rapid decrease in temperature in the lower layer of spent glaze can also contribute to the lack of pulp damage, due to high absorption of this substrate by the appropriate wavelength of 10.6 *μ*m CO_2_ laser [36]. The low energy density, used in this study, promoted thermal relaxation time of the deciduous enamel ranging between 1 and 60 *μ*s, and the pulse duration of the laser CO_2_ was 100 *μ*s. Esteves-Oliveira et al. 2009 [37] using energy density 0.3 J/cm^2^, similar to this study, were able to decrease enamel caries progression without causing surface and subsurface thermal damage.

CO_2_ laser action on primary and permanent enamel can be distinct, due to the differences between these substrates. The mineralization, calcium, and phosphorus percentage is lower in primary teeth than in permanent teeth [6]. The thickness of primary enamel is almost half of the permanent enamel that may have an influence on the demineralization [6] and may provide greater temperature rise when compared to permanent teeth, since thicker structures of enamel and dentin promote smaller temperature change [35, 38–41].

Carbonate content reduction on permanent enamel, promoted by CO_2_ laser irradiation [11], results in lower hydroxyapatite solubility. The increasing in crystals size [42], melting [23, 42], and fusion [11] of irradiated enamel have also reduced the enamel dissolution on permanent teeth against acid challenge, although melting of enamel tissues is not a necessity for laser radiation to inhibit caries formation in enamel [24]. In primary teeth, CO_2_ laser is also able to reduce carbonate content of enamel [9], which may have led to increased resistance to demineralization in this study.

It has been reported that, after a professional fluoride application, calcium fluoride (CaF_2_) is formed on enamel surface and fluoride is released to fluid phase. This effect promotes a consequent reduction of enamel demineralization. Also, a dose-response effect is observed between the concentration of CaF_2_, reservoirs on enamel and fluoride released, to “plaque fluid” and the subsequent inhibition of enamel demineralization [5]. The findings of this study have shown that topical application of APF in primary teeth is effective in the demineralization process and caries control [1, 2].

The amount of fluoride formed in the enamel depends on the concentration and the pH of the product applied and how long it remains in contact with the enamel [19]. Tenuta et al. 2008 [5] stated that the constant presence of fluoride in the oral cavity is more important than its concentration for the final enamel absorption. Thus, topical application of more acidic and concentrated fluoride compounds could provide effective protection against demineralization of tooth enamel or caries lesion formation [43], with higher incorporation of fluoride on enamel [19], however, no difference in fluoride uptake by enamel [19] was observed when fluoride was applied by one minute compared to four minutes.

In the present study, as CO_2_ laser was applied on previously demineralized primary enamel simulating a patient with high cariogenic challenge and high caries risk, it is difficult to make a direct comparison with these results to previous literary studies. Until now, there is no research that performed previously cariogenic challenge on primary teeth enamel, targeting the demineralization controlling and not preventing demineralization, having sound as substrate. Besides, the higher the demineralization is, the more difficult caries control becomes.

## 5. Conclusion

CO_2_ laser with *λ* = 10.6 *μ*m was effective in the control of demineralization on previously demineralized primary enamel, presenting some advantages on being a quick, comfortable, and simple method of applying, especially in children, considering the difficulty of using a fluoride. In this way, CO_2_ laser can be a resource in the control of caries lesions progression on primary teeth enamel.

## Figures and Tables

**Table 1:** Microhardness values (mean and standard deviation) according to the superficial treatments in different experimental groups (*P* = 0.03).

Treatment	Mean	Standard deviation
CO_2_ laser	324.99^a^	33.78
1.23% acidulated phosphate fluoride	309.30^a^	68.42
No treatment (control)	209.86^b^	67.03

Similar letters indicate statistical similarity.
